# Autoimmune origin for immune checkpoint inhibitor-diabetes revealed by deep immune phenotyping of the pancreas

**DOI:** 10.1136/jitc-2025-011818

**Published:** 2025-08-14

**Authors:** Zoe Quandt, Arabella Young, Graham Larson Barlow, Jennifer A Smith, Irina Kusmartseva, Shen Dong, Melanie R Shapiro, Jee Hye Kang, Jamie L Felton, Vinh Q Nguyen, Greg Szot, Assad A Hassoun, Ana Luisa Perdigoto, Kevan C Herold, Garry Nolan, Paul L Bollyky, Todd M Brusko, Maki Nakayama, Stewart Cooper, Mark S Anderson

**Affiliations:** 1 Department of Medicine, University of California San Francisco, San Francisco, California, USA; 2 Diabetes Center, University of California San Francisco, San Francisco, California, USA; 3 Huntsman Cancer Institute, University of Utah, Salt Lake City, Utah, USA; 4 Division of Microbiology and Immunology, Department of Pathology, University of Utah, Salt Lake City, Utah, USA; 5 Division of Infectious Diseases and Geographic Medicine, Department of Medicine, Stanford University School of Medicine, Stanford, California, USA; 6 Department of Pathology, Stanford University School of Medicine, Stanford, California, USA; 7 Department of Pathology, Immunology and Laboratory Medicine, University of Florida Diabetes Institute, Gainesville, Florida, USA; 8 Department of Pediatrics, Division of Pediatric Endocrinology and the Herman B. Wells Center for Pediatric Research, Indiana University School of Medicine, Indianapolis, Indiana, USA; 9 Center for Diabetes and Metabolic Diseases, Indiana University School of Medicine, Indianapolis, Indiana, USA; 10 Department of Surgery, University of California San Francisco, San Francisco, California, USA; 11 Department of Surgery, California Pacific Medical Center, San Francisco, California, USA; 12 Department of Surgery, Zheen International Hospital, Erbil, Iraq; 13 Department of Internal Medicine, Yale University, New Haven, Connecticut, USA; 14 Internal Medicine, VA Connecticut Healthcare System, West Haven, Connecticut, USA; 15 Department of Immunobiology, Yale University, New Haven, Connecticut, USA; 16 Department of Pediatrics, University of Florida, Gainesville, Florida, USA; 17 Department of Biochemistry and Molecular Biology, University of Florida, Gainesville, Florida, USA; 18 Department of Immunology and Microbiology, University of Colorado School of Medicine, Aurora, Colorado, USA; 19 Barbara Davis Center for Childhood Diabetes, University of Colorado School of Medicine, Aurora, Colorado, USA; 20 Liver Center, University of California San Francisco, San Francisco, California, USA; 21 Division of General and Transplant Hepatology, California Pacific Medical Center, San Francisco, California, USA

**Keywords:** Immune related adverse event - irAE, Diabetes, Immune Checkpoint Inhibitor

## Abstract

Immune checkpoint inhibitor-diabetes (CPI-D) is an acute and non-resolving immune-related adverse event (irAE) initiated primarily by disrupting the programmed death-1 (PD-1)/programmed death-ligand 1 (PD-L1) axis with monoclonal antibodies. A major limitation in understanding CPI-D is the lack of access to pancreatic tissue from patients experiencing this complication. We report a unique patient with no prior history of diabetes or autoimmune disease whose treatment with CPI for metastatic melanoma was complicated by CPI-D requiring insulin therapy. The patient then went on to develop pancreatic cancer. In the setting of the pancreatic cancer treatment, we were able to perform detailed single-cell RNA sequencing and immunophenotyping within the surgically resected pancreas. This revealed substantial lymphocytic infiltration associated with the islets, suggestive of an autoimmune rather than autoinflammatory mechanistic origin for CPI-D.

## Introduction

 Endocrinopathies constitute a significant immune checkpoint inhibitor (CPI)-related toxicity initiated in patients with cancer.^1 2^ Unlike many immune-related adverse events (irAEs), CPI endocrinopathies are largely irreversible, and there are no established interventions to prevent or attenuate their progression aside from hormone replacement. In fact, for CPI-diabetes (CPI-D), corticosteroids not only fail to provide benefit but can instead exacerbate hyperglycemia.^3^ CPI-D occurs in a small proportion of patients (between 0.2% and 2%) who are susceptible to hospitalization due to the acute onset, with 50–75% of patients in diabetic ketoacidosis (DKA) at diagnosis.^4^,^6^ Insulin deficiency is a hallmark of CPI-D; however, fewer than 50% of patients present with autoantibodies at the time of diagnosis.^1^ In addition, the disease is linked to type 1 diabetes (T1D) human leukocyte antigen (HLA)-risk alleles like DR4, which support an autoimmune origin of the disease.^1 4 7 8^ There has been a general lack of access to pancreatic tissue in patients affected by CPI-D, which has hindered our understanding of the pathogenesis of the disease. In this study, we report a case of CPI-D in which we accessed pancreatic tissue for spatial multiplex imaging as well as endocrine-specific islet isolation alongside tumor and peripheral blood mononuclear cells (PBMCs) to complete coordinated single-cell RNA sequencing (scRNA-seq) and T cell receptor (TCR) sequencing, which supports a T cell-mediated destruction of insulin-producing pancreatic beta cells.

## Case report

**Figure 1:**
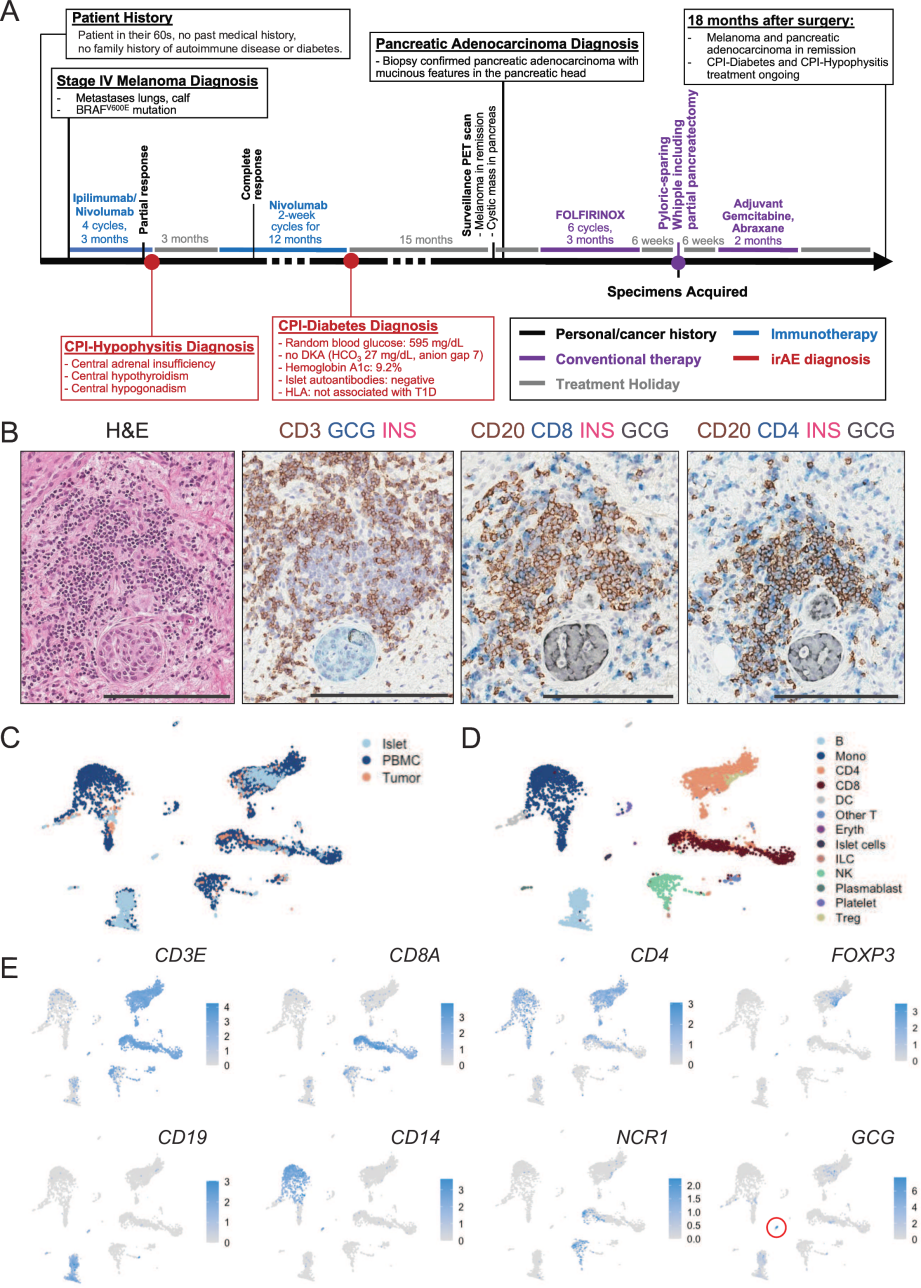
CPI-D is associated with T and B cell pancreatic infiltrate. (**A**) Schematic of case history relating to cancer diagnosis, cancer treatment, and development of irAEs, particularly CPI-D. (**B**) Histology of immune infiltrate surrounding an islet is shown by H&E (far left panel) and staining with glucagon (denoted as GCG for alpha cells), insulin (denoted as INS for beta cells), CD3, CD4 or CD8 (T cells), CD20 (B cells). Scale bar 200 µm. (**C**) Single-cell RNA sequencing of islets, PBMC and pancreatic tumor with (**D**) cluster identification through the Azimuth reference database. (**E**) Feature plot of immune markers used to identify cell types of interest is shown with the gene expression in blue (logCPM), red circle denotes *GCG*-expressing islet cells. CPI, immune checkpoint inhibitor; CPI-D, CPI-diabetes; DC, dendritic cell; DKA, diabetic ketoacidosis; Eryth, erythrocyte; FOXP3, forkhead box protein P3; HLA, human leukocyte antigen; ILC, innate lymphoid cell; INS, insulin; irAE, immune-related adverse event; Mono, monocyte; NCR1, natural cytotoxicity triggering receptor 1; NK, natural killer; PBMC, peripheral blood mononuclear cell; PET, positron emission tomography; Treg, regulatory T cell; T1D, type 1 diabetes.

A patient in their late 60s with no prior medical history aside from BRAF-mutant metastatic melanoma was treated with combination ipilimumab (an anti-cytotoxic lymphocyte associated-4 monoclonal antibody) and nivolumab (an anti-programmed death-1 (PD-1) monoclonal antibody) (figure 1A). After 4 cycles given every 3 weeks, the patient displayed a robust but not yet complete tumor response alongside development of hypophysitis with secondary adrenal insufficiency, hypothyroidism, and hypogonadism. The patient was treated with appropriate hormone replacement therapies prior to resuming nivolumab monotherapy every other week. Approximately 3 months later (ie, 6 months after CPI initiation), the patient achieved a complete response for the melanoma and continued receiving nivolumab every other week for the next year.

Approximately 6 weeks after the last dose of nivolumab, 18 months after CPI initiation, the patient developed polydipsia, polyuria, orthostasis, and steatorrhea. At hospitalization, the patient was found to have a blood glucose of 595 mg/dL (normal reference range 65–139 mg/dL) without DKA. The patient was initially prescribed metformin, but with ongoing elevated glucose over 400 mg/dL, they were transitioned to basal-bolus insulin and diagnosed with CPI-D. The patient was also started on pancreatic enzyme replacement for steatorrhea and presumed exocrine pancreas insufficiency. One week after this initial presentation, the hemoglobin A1c was 9.2% (normal reference range 4–5.6%). One year after CPI-D diagnosis, islet cell autoantibodies testing against glutamic acid decarboxylase 65 (GAD65), zinc transporter 8, islet antigen 2, and insulin were negative. A stimulated C-peptide was <0.10 nmol/L (reference range 0.80–3.85 nmol/L) for a blood glucose of 230 mg/dL through a pseudo mixed-meal tolerance test, consistent with non-resolving CPI-D at 1 year from CPI-D diagnosis. The HLA type (HLA-A1/A2, B44/B44, DR*11:01/DQ*7.5, DR*13/DQ*6.3) is not considered high risk for spontaneous T1D.^9^

Fifteen months after CPI-D diagnosis, and with melanoma remission, a routine positron emission tomography (PET) scan identified a cystic mass in the head of the pancreas and a subsequent biopsy confirmed pancreatic adenocarcinoma. The patient completed 6 cycles of chemotherapy (FOLFIRINOX), leading to a decrease in size of the pancreatic adenocarcinoma, followed by a pyloric-sparing Whipple surgery. Pancreatic tissue, tumor and blood were retrieved at the time of surgery which prompted an array of downstream assays (online supplemental figure S1A). Pathology showed negative surgical margins and no positive lymph nodes (of 16 tested). Eighteen months after surgery, the patient had no evidence of recurrent pancreatic cancer or melanoma but continued basal-bolus insulin replacement therapy.

## Methods

The patient and care team identified the unique opportunity afforded by these diagnoses to study the pancreatic microenvironment in CPI-D. Pancreatic tissue from this patient was recovered following a Whipple procedure and used for multiple downstream assays (online supplemental figure S1A). Immunohistology and co-detection by indexing (CODEX) multiplexed imaging were used to identify immune, endocrine, and microenvironmental markers from defined pancreatic regions (online supplemental figure S1B, C).^10^ Pancreatic islets were also isolated by enzymatic digestion from a proportion of non-cancerous pancreas by the University of California San Francisco (UCSF) Clinical Islet Core. All cells retrieved from the islets were processed for downstream combined scRNA-seq and TCR sequencing and compared with PBMCs drawn at the time of surgery as well as flow cytometry sorted viable cells from collagenase/DNase digested pancreatic tumor. Cells were analyzed using Seurat,^11^ and clusters were then characterized using the Azimuth reference datasets^12^ for PBMCs and islets followed by manual validation. TCR sequences were compared against established TCR databases for spontaneous T1D from Network for Pancreatic Organ Donors with Diabetes (nPOD) and the Manually Curated Pathology-Associated T Cell Receptor (McPAS-TCR) database through comparison of complementarity determining region 3 beta (CDR3β) sequences.^13^,^17^ For the McPAS-TCR database,^13^ which is a manually curated database of disease-derived TCR sequences for CD4^+^ and CD8^+^ T cells, matched CDR3βs were allowed a maximum Levenshtein distance (LD) of 1. From the nPOD program, which includes TCR sequences from CD4^+^ and CD8^+^ T cells, only exact CDR3β matches were included.^16 17^ Additional methodological details are supplied in the online supplemental appendix.

## Results

Histological analysis of the pancreas identified that the few remaining islets were primarily devoid of insulin-producing beta cells but did have glucagon positivity (figure 1B). Surrounding the islets were lymphoid aggregates consisting of CD20+ B cells along with CD3+CD4+ and CD3+CD8+ T cells (figure 1B). This was consistent with the immune profile from the isolated islets as determined by scRNA-seq, in which the majority of the immune infiltrate was lymphocytic in nature. The most frequent immune cell components by scRNA-seq displayed transcriptional expression of *CD19* (B cells), as well as *CD3E* alongside either *CD8A* (CD8^+^ T cells) or *CD4* (CD4^+^ T cells) (figure 1C–E and online supplemental figure S2). Endocrine cells were also identified by scRNA-seq, but similar to the histological findings, this was restricted to glucagon (*GCG*)-expressing cells (figure 1E and online supplemental figure S2). Previous studies on the pathology of spontaneous T1D have also shown similar patterns with complete loss of insulin-expressing beta cells in pancreatic islets and associated immune infiltrate.^18^ We also identified comparable features from our patient with CPI-D in patients with spontaneous T1D from the nPOD tissue bank (online supplemental figure S3).

**Figure 2:**
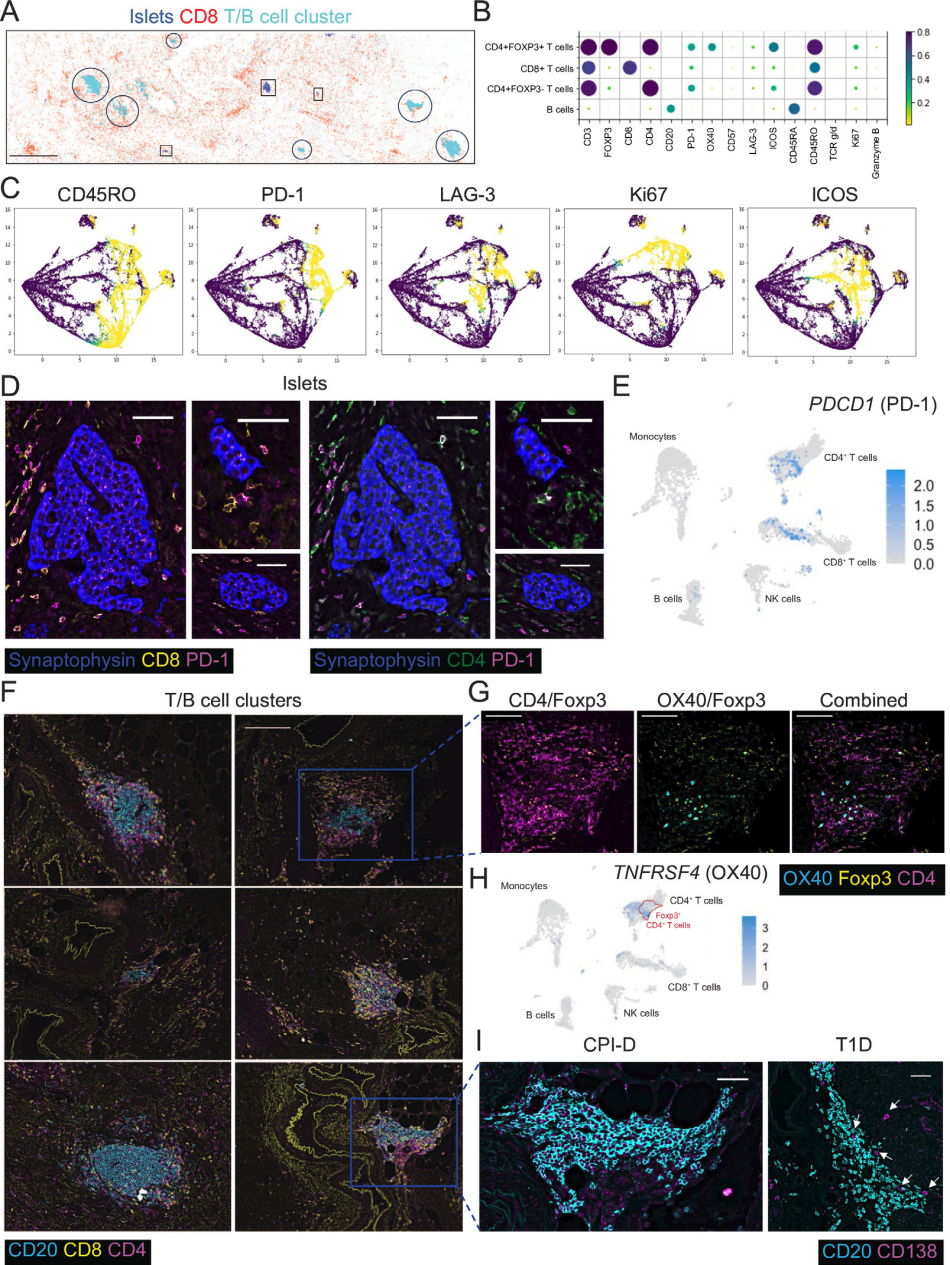
Prominent T cell activation and lack of antibody-producing plasma cells are characteristics of CPI-D. (**A**) Overview of pancreatic tissue from the patient with CPI-D with the distribution of islets (in boxes), T/B cell clusters (in circles), and CD8+ T cells (in red) identified by CODEX. Scale bar 1 mm. (**B**) Mean frequencies of immunomodulatory markers expressed in CD8+ T cells, CD4+FOXP3- T cells, CD4+FOXP3+ T cells, and B cells in the pancreas from the patient with CPI-D. (**C**) Co-expression of immunomodulatory markers in pancreatic CD8+ T cells is demonstrated by UMAP. (**D**) Expression of PD-1 in T cells associated with islets from a patient with CPI-D. Scale bar shown in each individual panel is 50 µm. (**E**) Gene expression of *PDCD1* (the PD-1 encoding gene) from PBMCs, islets and pancreatic tumor cells by scRNA-seq; cluster annotation detailed in figure 1D. (**F**) T/B cell clusters within the pancreas are composed of CD20+ B cells, CD4+ T cells and CD8+ T cells. Representative scale bar 200 µm is consistent across all regions shown. (**G**) OX40 is expressed in CD4+FOXP3+ T cells in T/B cell clusters. Scale bar shown in each individual panel is 100 µm. (**H**) Gene expression of *TNFRSF4* (the OX40 encoding gene) from PBMCs, islets and pancreatic tumor by scRNA-seq and comparison to the *FOXP3* gene expression region outlined in red and further detailed in figure 1E; cluster annotation detailed in figure 1D. (**I**) CD138 expression in T/B cell clusters in the pancreas of an individual with CPI-D or T1D. Scale bar shown in each individual panel is 50 µm. CODEX, co-detection by indexing; CPI-D, immune checkpoint inhibitor-diabetes; FOXP3, forkhead box protein P3; ICOS, inducible T cell costimulator; Ki67, kiel 67; LAG-3, lymphocyte-activation gene 3; NK, natural killer; PBMC, peripheral blood mononuclear cell; PD-1, programmed death-1; scRNA-seq, single-cell RNA sequencing; T1D, type 1 diabetes; UMAP, Uniform Manifold Approximation and Projection.

We next used CODEX to perform in-depth phenotyping of immune cells alongside their spatial distribution in the pancreas. We identified islets, clusters of CD3+ T cells and CD20+ B cells (“T/B cell cluster”), and CD8+ T cells (figure 2A). We then characterized the expression of functional markers on each major T cell subset (CD4+FOXP3−, CD4+FOXP3+ and CD8+) and B cells (CD20+) (figure 2B). The majority of all three T cell subsets expressed the memory marker CD45RO+ and the next most frequently expressed marker in the three T cell subsets was PD-1. OX40 and ICOS (inducible T cell costimulator) were primarily expressed by CD4+FOXP3+ T cells (figure 2B). UMAP (Uniform Manifold Approximation and Projection) analysis of the CD8+ T cells, derived from CODEX data, demonstrated that PD-1-expressing cells co-expressed LAG-3 (lymphocyte-activation gene 3), Ki67 (kiel 67) and ICOS (figure 2C). As it is thought that PD-1 signaling provides a critical tolerance mechanism in preventing an immune reaction against islet cells,^7^ we next assessed the expression of PD-1 by islet-associated immune infiltrate. Both CD4+ and CD8+ T cells surrounding the islets, as measured with CODEX, and islet-associated immune infiltrate, as analyzed with scRNA-seq, expressed PD-1 (figure 2D, E).

Within the T/B cell clusters identified in the pancreatic tissue with CODEX (figure 2A and F), OX40 expression was present and largely restricted to FOXP3-expressing CD4+ T cells (figure 2G and online supplemental figure S4A). Significantly enriched *TNFRSF4* (OX40) expression was also identified in FOXP3^+^CD4^+^ T cells compared with other islet-infiltrating T cell compartments by scRNA-seq (figure 2H). OX40 was also expressed by CD4+FOXP3+ T cells not in T/B cell clusters in the CPI-D pancreas (online supplemental figure S4B). However, there was an 8.4-fold enrichment in the presence of CD4+FOXP3+ T cells localized to the T/B cell clusters. OX40 was also expressed at comparable levels in CD4+FOXP3+ T cells in the pancreas of individuals with spontaneous T1D (online supplemental figure S4C, D). In contrast to the CPI-D case, the presence of CD4+FOXP3+ T cells was rare in T/B cell clusters from spontaneous T1D individuals, detected in only one of eight pancreata, which is consistent with previous assessments in tertiary lymphoid structure (TLS) in spontaneous T1D.^19^

In addition, CD138+ cells, indicative of antibody-producing plasma cells, were not detected in the T/B cell clusters nor were CD138+ cells detected throughout the CPI-D pancreatic tissue, which may be consistent with the islet-antigen associated autoantibody negative status of the patient (figure 2I). In contrast, over 90% of patients with spontaneous T1D are autoantibody positive at diagnosis and many display evidence of CD138-expressing plasma cells in T/B cell clusters within the pancreas (figure 2I and online supplemental figure S5A).^19^,^22^ Comparison of B cells found within the islets and PBMCs showed enrichment towards different subsets, with naïve B cells being more common in the periphery than the islets (online supplemental figure S5B). Islet-derived B cells had increased expression of features associated with the transcriptional profile of germinal center B cells (*CD22, MARCKSL1*), which may be due to their localization in the tissue (online supplemental figure S5C). This also suggests their role in antigen presentation, further enforced by the increased *CD83* expression (which supports T-B cell interactions in activated B cells) (online supplemental figure S5C).^23^ It is difficult to discern which gene expression features are unique to CPI-D and which are consistent with normal pancreas, but this profiling provides the backbone for further integrated studies.

**Figure 3:**
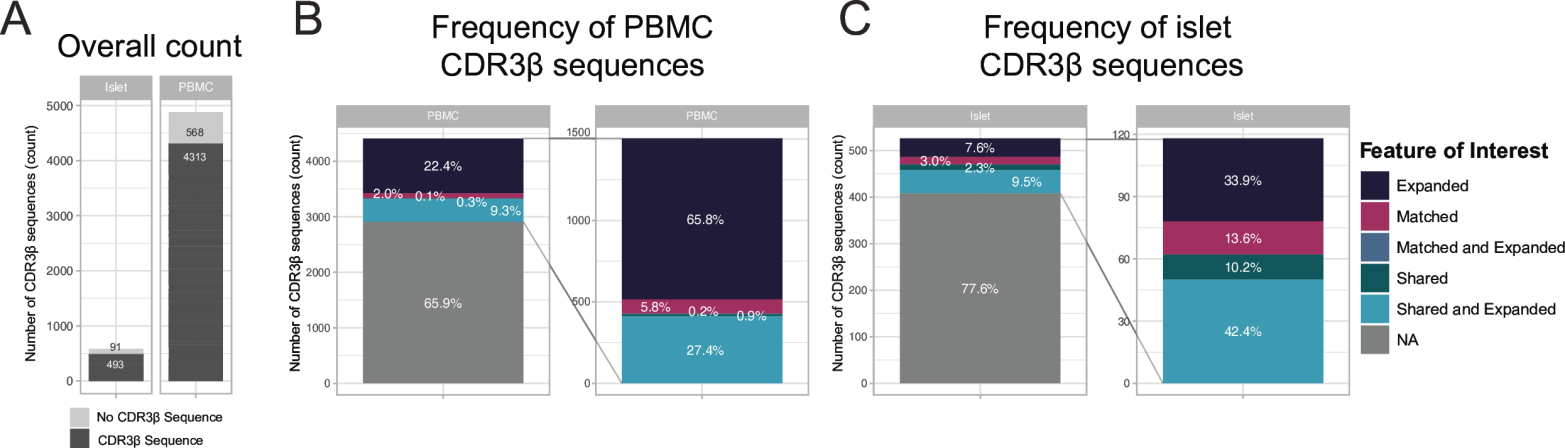
CDR3β sequences are expanded, shared between compartments, and matched to spontaneous T1D subjects. (**A**) Number of T cells with a CDR3β sequence isolated from islets and PBMCs relative to the number of T cells without CDR3β sequences. Number of T cells with CDR3β sequences from (**B**) PBMCs and (**C**) islets alongside the frequency (percent, in white) of the features of interest, including sharing across compartments (PBMCs, islet, pancreatic tumor), expansion within compartments (≥2 of the same CDR3β sequence), and identification of matched CDR3β sequences (either exact or within 1 LD) from subjects with spontaneous T1D within the nPOD database and the publicly available McPAS-TCR database. CDR3β, complementarity determining region 3 beta; McPAS-TCR, Manually Curated Pathology-Associated T Cell Receptor database; nPOD, Network for Pancreatic Organ Donors with Diabetes; PBMC, peripheral blood mononuclear cell; T1D, type 1 diabetes.

Using the scRNA-seq paired with TCR sequencing from the islet, pancreatic tumor, and PBMC of the patient with CPI-D, we assessed the expanded CDR3β regions and whether they were shared across different organs (figure 3 and online supplemental table S1). There were 37 distinct CDR3β sequences in the islets, 157 in the PBMCs, and 22 in the pancreatic tumor that were either shared across tissue types, expanded within a compartment or both after annotation of T cell type (figure 3). Those with more than two cells identified by scTCR-seq and that are shared across more than one compartment are listed (online supplemental table S1). Shared and expanded CDR3β sequences were particularly prominent within the CD8^+^ T effector memory cells (online supplemental figure S6A, B). We next used existing TCR databases from McPAS-TCR and the nPOD program to determine whether CDR3β sequences from subjects with spontaneous T1D matched to those isolated from the patient with CPI-D.^13 16 17 24^ Matched CDR3β sequences from both databases were identified in the islets, PBMC, and pancreatic tumor from the patient with CPI-D (figure 3B, C and online supplemental figure S6C, online supplemental table S1). From the McPAS-TCR database, there were 7 distinct CDR3β sequences that were exact matches and 83 CDR3β sequences within 1 LD. Intriguingly, 3 of these CDR3β sequences were annotated in the McPAS-TCR database to have proposed antigen specificity to insulin precursor and GAD65, suggesting potential islet reactivity (online supplemental table S1).^13^ From the nPOD database, there were 22 distinct CDR3β sequences that were exact matches, including 8 CDR3β sequences that were also shared with McPAS-TCR (either exact or within 1 LD) (online supplemental table S1). These public CDR3β sequences suggest possible sharing of antigenic targets common to both CPI-induced and spontaneous forms of autoimmune diabetes.

## Discussion

CPI-D is a non-resolving irAE primarily initiated by disruption to the PD-1/programmed death-ligand 1 (PD-L1) immunoregulatory axis.^7^ Although a number of factors point to an autoimmune origin of the disease, there has been little published data on tissue histology from affected patients, as access to the pancreas of individuals with CPI-D is extremely rare. Unlike many other irAEs, exposure to immunosuppressive agents or CPI treatment discontinuation does not lead to recovery of endocrine pancreatic function, highlighting the need for in-depth profiling of the immune response to identify the mechanism for CPI-D initiation and new therapeutic targets. This unique case represented an opportunity to perform high-dimensional immune profiling from a biopsy of the pancreas from a living individual with CPI-D.

Our results support a beta cell-specific autoimmune etiology of CPI-D and highlight specific immune cell subsets that may be critical to the development of this rare complication. In a small number of prior reports from CPI-D affected pancreata (including one surgical and three autopsy cases),^25 26^ similar features with our case were observed, such as few remaining insulin-positive beta cells, residual alpha cells, and a T-cell predominant immune infiltrate. We were able to further characterize that T cells from the pancreas of the patient with CPI-D often shared a considerable number of activation and functional markers, including PD-1-expressing CD4^+^ and CD8^+^ T cells surrounding the remaining islets, which may be triggered to initiate an autoimmune response following CPI exposure. An increase in infiltrating macrophages in the pancreas of patients with CPI-D relative to patients with CPI-treated cancer who did not develop diabetes has also been previously reported.^25^ In our patient, macrophages were present but were a minor component of the islet-associated immune infiltrate. The presence of B cells has not been previously reported in CPI-D, but they represented a major component of the immune infiltrate we observed and are common in early childhood insulitis.^18^ Despite the abundance of B cells within this pancreas, this patient was negative for islet autoantibodies associated with T1D 1 year after CPI-D diagnosis, close to when the pancreas sample was collected. This also reflected the lack of CD138^+^ plasma cells within the CPI-D pancreas.^18 21^ The temporal dynamics of the immune response following CPI-D diagnosis remain unclear, and it will be important to build a profile of both longitudinal changes and the heterogeneity of the immune response in the pancreas as cases continue to emerge.

The presence of T/B cell clusters was associated with both islets and non-cancerous but irAE-affected pancreas. The punctate distribution of these lymphocytic aggregates in the pancreas may also represent areas where islets were co-located and destroyed during CPI-D pathogenesis. TLS has been found in CPI-induced acute interstitial nephritis (AIN)^27^ as well as age-associated preclinical models of CPI-induced multiorgan irAEs,^28^ suggesting a possible common irAE mechanism that should be explored more broadly in other tissues. Additionally, TLS have been seen in the progression to spontaneous T1D in both preclinical and clinical disease and their manipulation has even been able to prevent T1D in a preclinical model.^19 29^ Inhibiting TLS formation using B cell depleting agents, such as rituximab (an anti-CD20 monoclonal antibody),^30^ may also assist in limiting irAE progression. This therapeutic modality has received increasing attention as a possible strategy to inhibit irAEs, particularly in severe or steroid-refractory cases.^31 32^ Rituximab has also been shown to transiently preserve beta cell function in a subset of individuals with recent-onset spontaneous T1D,^33 34^ potentially temporarily dampening the immune response but has also been shown not to reset defective tolerance checkpoints in animal models.^35^ In some irAEs, this temporary dampening may be sufficient to halt the irAE; however, a greater understanding of the heterogeneity relating to the presence and function of B cells in CPI-D, both in the pancreas and periphery, is needed. We observed gene expression features relating to germinal center B cells without evidence of plasma cell accumulation in the CPI-D pancreas. This may be consistent with preclinical mouse studies that identified that PD-1^+^ T follicular helper cells interact with PD-L1 in B cells to support plasma cell formation and in the absence of either PD-1 or PD-L1, the development of long-lived plasma cells was decreased.^36 37^ Depleting B cells from solid tumors in preclinical models has shown minimal impact on CPI treatment efficacy in certain cancers,^38^ which may highlight an opportunity to perform prophylactic B cell depletion in patients with high risk for irAEs mediated by B cells in at least some tumor types.

OX40 was strongly expressed by the CD4^+^FOXP3^+^ T cells in the CPI-D pancreas, especially within the T/B cell clusters. Interestingly, in CPI-induced AIN, which also has a strong presence of TLS, OX40 expression was found to be significantly increased in this form of kidney injury compared with those that do not form TLS.^27^ OX40 expression has been shown to both inhibit and enhance regulatory T cell-mediated suppression^39 40^ with an agonistic OX40 antibody shown to reduce autoimmune diabetes incidence through an antigen-specific mechanism in non-obese diabetic mice.^41^ More recently, agonistic OX40 agents have been deployed in early-stage clinical trials either alone or in combination with CPI treatment in solid tumors.^42 43^ Given that OX40 has been identified as potentially enriched in certain types of irAE-affected tissue, it will be important to maintain a high level of clinical vigilance to determine whether agonists of this pathway can alter the severity and type of irAEs that develop in patients with cancer.

While there is heterogeneity in the presence of clinical characteristics that underlie CPI-D development,^1^ this patient does have some features that make their case atypical. It is not uncommon to be islet autoantibody negative at diagnosis or to have concurrent exocrine pancreatic insufficiency, but this patient’s lack of an HLA type associated with T1D risk is rare.^4^ Furthermore, while there was no imaging evidence of their pancreatic cancer duringmelanoma treatment, it is possible that the patient had microscopic disease already present that triggered an autoimmune response leading to CPI-D, with some evidence that presence of tumor can heighten risk for irAEs in the same tissue.^44^ Alternatively, the inflammation initiated by CPI-D may have reduced immune surveillance that led to the development of pancreatic cancer. Despite the temporal limitations and differences between our patient and ‘prototypical’ CPI-D, this case offers a critical lens to assess the pancreatic tissue microenvironment and shows the presence of a targeted immune response involving both B and T cells, similar to that seen in a proportion of T1D cases.^18^ Furthermore, the presence of islet-infiltrating T cells with CDR3β sequences that overlap with those identified in individuals with T1D suggests the potential for shared islet antigen specificity between the two autoimmune diabetes types. Our next step will be to determine TCR antigen recognition between clones identified in both the CPI-D islet and T1D subjects as well as across different tissue compartments, such as the islet and pancreatic tumor samples, to provide the unique ability to assess antigen specificity and cross-reactivity in CPI-D. Additionally, it is of interest to establish whether TCR sequences recur across patients with CPI-D, as well as other types of autoimmune diabetes, and whether this can be identified in the peripheral blood and may be used as a non-invasive signature for susceptibility to CPI-D.

While potential therapeutic options to postpone or prevent T1D are emerging, with most notably the Food and Drug Administration approval of teplizumab (an anti-CD3 monoclonal antibody),^45 46^ there is still much to be done in this area, as there are no known therapeutic options available for CPI-D aside from insulin administration. Further studies of the immune microenvironment in CPI-D pancreata can help to provide mechanistic insights to inform choices for therapeutic trials; however, this will continue to be challenging due to the limited accessibility of pancreatic tissue in living patients, particularly at the time of CPI-D diagnosis, and the ineligibility of deceased patients with cancer for organ donation to organ procurement organizations that contribute to research.^47^ This represents a need for coordinated research efforts, as here, that incorporate multiple disciplines to improve mechanistic understanding of these complex and often heterogeneous diseases. Therapeutic options must be considered carefully, to enable opportunities to uncouple immunotoxicity from antitumor immunity without risk of exacerbating tumor growth or recurrence.^48^ This highlights the critical nature of studies that delineate the landscape of irAE-affected tissue to complement the growing understanding of immunoregulatory mechanisms in the tumor microenvironment.

## Supplementary material

10.1136/jitc-2025-011818 online supplemental file 1

10.1136/jitc-2025-011818 online supplemental file 2

10.1136/jitc-2025-011818 online supplemental file 3

10.1136/jitc-2025-011818 online supplemental file 4

10.1136/jitc-2025-011818 online supplemental file 5

10.1136/jitc-2025-011818 online supplemental file 6

10.1136/jitc-2025-011818 online supplemental file 7

10.1136/jitc-2025-011818 online supplemental file 8

## Data Availability

Data are available upon reasonable request.
