# Supplementary material for: Autoimmune origin for immune checkpoint inhibitor-diabetes revealed by deep immune phenotyping of the pancreas
Source: J Immunother Cancer. 2025 Aug 14;13(8):e011818. doi: 10.1136/jitc-2025-011818 (PMC12359507; doi:10.1136/jitc-2025-011818)
Supplement: online supplemental file 1 [file jitc-13-8-s001.docx]

**Supplementary Table 1. Selected CDR3β sequences shared between PBMC, islet and tumor, expanded, or matched to T1D TCR databases.**

| **Azimuth cluster** | **Cell Count by Compartment** | | | **CDR3β sequence** |
| --- | --- | --- | --- | --- |
|  | **PBMC** | **Tumor** | **Islet** |  |
| **Expanded (2 or more) and shared between compartments** | | | | |
| CD4 | 2 | 2 |  | CSANKQGAEAFF |
| CD8 | 209 | 2 |  | CASRQGQHTEAFF |
|  | 145 | 6 | 12 | CASSQSPGGLAFF |
|  | 3 | 3 | 9 | CASGSGQPNTEAFF |
|  | 8 | 2 | 9 | CASSQDRTGSYGYTF |
|  | 11 |  | 4 | CASSPYVRDRGEETQYF |
|  | 6 |  | 4 | CASTIGPWGPEAFF |
|  | 22 | 2 |  | CASSQRTSGGSYNEQFF |
|  | 2 |  | 8 | CASSYSGTGGELFF |
|  | 2 |  | 2 | CSATRQGQTQYF |
| **Expanded and matched to either nPOD or McPAS-TCR database**  **(exact or within 1 LD)** | | | | |
| CD8 | 3 |  |  | CASSLGNTEAFF ^ |
| Other T |  | 2 |  | CASSQERGGQETQYF |
| **Matched to both nPOD and McPAS-TCR database**  **(exact or within 1 LD)** | | | | |
| CD4 | 1 |  |  | CASSLGSTDTQYF ^ |
|  | 1 |  |  | CASSLGETQYF * ^#^ ^ |
|  | 1 |  |  | CASSEGNYGYTF ^ |
|  | 1 |  |  | CASSSSTDTQYF |
|  | 1 |  |  | CASSPTGNSNQPQHF |
| CD8 | 1 |  |  | CASSLGETQYF * ^#^ ^ |
|  | 1 |  |  | CASSIRSSYEQYF ^ |
|  | 1 |  |  | CASSLGSGNTIYF ** ^ |
|  |  |  | 1 | CASSEGYGYTF |
| **Matched to McPAS-TCR database (exact)** | | | | |
| CD4 | 1 |  |  | CASSLGGYEQYF |
|  | 1 |  |  | CASSLTYEQYF |
|  | 1 |  |  | CASSFGQETQYF |
|  | 1 |  |  | CASSLETQYF* |
| **Matched to nPOD database (exact)** | | | | |
| CD4 | 1 |  |  | CASRDSNQPQHF |
|  | 1 |  |  | CASSQDRDTQYF |
|  | 1 |  |  | CASSLGQGNTEAFF |
|  | 1 |  |  | CASRGDWGSQPQHF ^##^ |
|  | 1 |  |  | CASSLVGGEQFF |
|  | 1 |  |  | CASSPRTDTQYF |
|  | 1 |  |  | CASSSRGTEAFF |
|  | 1 |  |  | CASSVGQGSYNEQFF |
|  | 1 |  |  | CASSQDHSGANVLTF |
|  |  |  | 1 | CASSLGQGDTEAFF |
| CD8 | 1 |  |  | CASRGDWGSQPQHF ^##^ |
| Treg |  |  | 1 | CASGNSNQPQHF |

^ Match to McPAS-TCR database is within 1 LD.

* Proposed antigen recognition to insulin precursor A1-A15 within McPAS-TCR database.

** Within 1 LD of a CDR3β that has proposed antigen recognition to GAD65 within McPAS-TCR database.

^#^ Clustering identified these two cells as distinct, annotated as a CD4^+^ T cell and a CD8^+^ T cell, respectively. These two cells have distinct TRBV, but the same TRBJ.

^##^ Clustering identified these two cells as distinct, annotated as a CD4^+^ T cell and a CD8^+^ T cell, respectively. It should be noted that the TRBV, TRBJ are common to both cells.
