# Supplementary material for: Autoimmune origin for immune checkpoint inhibitor-diabetes revealed by deep immune phenotyping of the pancreas
Source: J Immunother Cancer. 2025 Aug 14;13(8):e011818. doi: 10.1136/jitc-2025-011818 (PMC12359507; doi:10.1136/jitc-2025-011818)
Supplement: online supplemental file 2 [file jitc-13-8-s002.docx]

**Supplementary Appendix**

**Materials and Methods**

**Spontaneous T1D Pancreatic Tissue**

Transplant-quality pancreas was recovered from organ donors with type 1 diabetes (T1D) by the Network for Pancreatic Organ Donors with Diabetes (nPOD) program (www.jdrfnpod.com) according to established protocol and procedures(1,2), as approved by the University of Florida Institutional Review Board (201600029), the United Network for Organ sharing (UNOS), and according to federal guidelines with informed consent obtained from each donor’s legal representative. Cases of nPOD donor’s utilized in this study include 6228 (RRID:SAMN15879284), 6520 (RRID:SAMN18053203), 6534 (RRID: SAMN18242778) as well as additional cases previously analyzed by co-detection by indexing (CODEX) in Barlow *et al*. (3)*.*

**Histology and Immunohistochemistry**

Histology

Normal pancreatic tissue was taken during a partial pancreatectomy as part of a Whipple procedure for the immune checkpoint inhibitor-diabetes (CPI-D) patient and was embedded for formalin-fixed paraffin embedded (FFPE) tissue examination. Clinical excess tissue was made available from California Pacific Medical Center (CPMC). Pancreata from nPOD organ donors were centrally processed at the nPOD Organ Processing and Pathology Core, University of Florida. Each pancreas was divided into a head, body, and tail region. Each region was subjected to serial transverse sectioning and used for preparation of FFPE tissue blocks. Pancreatic tissue from both the CPI-D patient and organ donors from the nPOD program were cut at 4 µm thickness. Hematoxylin and eosin (H&E) staining was performed.

Immunohistochemistry

For triple and quadruple-stained slides, FFPE pancreatic tissue sections were deparaffinized and rehydrated with serial passage through changes of xylene and graded ethanol. All slides were then subjected to heat induced antigen retrieval in Borg Decloaker RTU (BioCare Medical, Pacheco, CA) followed by 3% H_2_O_2_. After washing, tissues were blocked with Background Sniper (BioCare Medical) prior to staining.

For triple-stained slides, blocked slides were incubated for 20 minutes at room temperature (RT) with the first primary antibody cocktail consisting of antibodies against CD3 (rabbit polyclonal anti-CD3, 1:100 dilution, Agilent Technologies, RRID:AB_2335677) and glucagon (mouse monoclonal anti-glucagon, 1:1000 dilution, Abcam, RRID:AB_297642), prior to being washed and incubated with MACH 2 Double Stain Kit 1 (BioCare Medical) for 20 minutes at room temperature (RT). Slides were then washed and developed using DAB and Ferangi Blue Chromogen solutions (both BioCare Medical), then subjected to a second round of heat-induced antigen retrieval in Borg Decloaker TRU followed by 3% H_2_O_2_. After washing, slides were again blocked with Background Sniper, washed, and incubated with the second primary antibody cocktail consisting of an insulin antibody (rabbit monoclonal anti-insulin, 1:2000 dilution, Abcam, RRID:AB_2716761) for 30 minutes at RT. After washing, slides were incubated with MACH 2 Double Stain Kit 2 for 30 minutes at RT, washed, and developed with Warp Red Chromogen Solution (BioCare Medical) followed by Hematoxylin counterstain.

For quadruple-stained slides, blocked slides were incubated with first the primary antibody against CD20 (rabbit monoclonal anti-CD20, 1:200 dilution, Abcam, RRID:AB_1640323) for 20 minutes at RT, then washed and incubated with MATCH4 polymer (Biocare Medical) for 20 minutes at RT. Slides were washed and developed using DAB Chromogen solution, then subjected to a second round of heat-induced antigen retrieval in Borg Decloaker TRU followed by 3% H_2_O_2_. After washing, tissue sections were blocked with Background Sniper following a 1-hour incubation with a second primary antibody (either rabbit monoclonal anti-CD4, 1:50 dilution, Abcam, RRID:AB_2750883 or mouse monoclonal anti-CD8, 1:100 dilution, Agilent Technologies, RRID:AB_2075537). After washing, slides were incubated with MACH 2 Double Stain kit 1 for 30 minutes at RT. Slides were washed and developed using Ferangi Blue Chromogen solution, then subjected to a third round of heat-induced antigen retrieval in Borg Decloaker TRU followed by 3% H_2_O_2_. After washing, tissue sections were blocked with Background Sniper followed by a 30 minute incubation with a cocktail of primary antibodies for glucagon (mouse monoclonal anti-glucagon, 1:1000 dilution, Abcam, RRID:AB_297642) and insulin (rabbit monoclonal anti-insulin, 1:2000 dilution, Abcam, RRID:AB_2716761). After washing, slides were incubated with Mach 2 Double Stain Kit 2 for 30 minutes at RT, washed again and developed with Warp Red and Deep Space Black Chromogen solutions (Biocare Medical) followed by Hematoxylin counterstain.

All experiments included human pancreatic tissue sections from non-diabetic donors as a positive control for insulin and glucagon staining and human spleen tissue sections as a positive control for immune cells. Appropriate IgG isotype control abs were used as a negative control to validate experimental results.

Image acquisition

Following H&E, triple-, or quadruple-IHC staining, whole slides were scanned at an absolute magnification of 20x using an Aperio CS2 Scanscope (Leica/Aperio, Vista, CA). Images were taken using Aperio ImageScope v. 12.4.3.

**Co-detection by indexing (CODEX)**

CODEX data collection

CODEX with FFPE-preserved tissue was performed as previously described(4–6). A Keyence BZ-X710 fluorescence microscope with a CFI Plan Apo λ 20x/0.75 objective (Nikon) was used in “High resolution” mode, resulting in a final resolution of 0.37744 μm/pixel. Data was processed using the CODEX Toolkit uploader [github.com/nolanlab/CODEX](7). CellSeg(8) was used for cell segmentation using the Draq 5 nuclear channel.

Cell type annotation

The marker expression was z-normalized and cell types were annotated with Uniform Manifold Approximation and Projection (UMAP) using the UMAP-learn python package (https://umap-learn.readthedocs.io/en/latest/) and Leiden clustering using the leidenalg python package (https://github.com/vtraag/leidenalg). First, major cell types were identified using the following markers: Chromogranin A (endocrine cells), CD31 (endothelium), MUC-1 (ducts), Cytokeratin and Na^+^K^+^-ATPase (epithelium), alpha-Smooth Muscle Actin (smooth muscle), CD45 (immune), Synaptophysin (endocrine or nerves), Podoplanin (lymphatics), S100A6 (stroma), and blank cycles. Immune cells were further sub-clustered to identify CD8+ T cells, CD4+FOXP3- T cells, CD4+FOXP3+ T cells, B cells, myeloid cells, and neutrophils.

Quantification of marker expression

Due to the low expression of many T cell markers, a neural network was trained to detect expression on a given cell using the raw image from the respective channel rather than the mean intensity within the cell’s segmentation mask. A neural network using the ResNet architecture was trained on images manually labelled by an immunologist familiar with the expression patterning of each marker. In total, 3963 cells with 15 markers each were labelled to produce a full dataset of 59,445 cell images. These images were split into training, validation, and test splits of 60%, 15%, and 25% respectively. The 25th, 50th, and 75th percentiles for the Recall across the 15 tested markers were 55%, 65%, and 80% respectively. The 25th, 50th, and 75th percentiles for the Precision across the 15 tested markers were 78%, 82%, and 87% respectively. The neural network’s predictions were used for Figures 2B-C. For comparison of marker expression in immune cells from the pancreas of CPI-D and autoantibody positive or spontaneous T1D individuals in Figure S4, mean fluorescence intensity (MFI) in CD4+FOXP3+ T cells, CD4+FOXP3- T cells, CD8 T cells, and B cells was used.

Identification of islets and T/B cell clusters

A previously described algorithm(5,9) was used to identify the islets and T/B cell aggregates within the tissue. Briefly, cells were clustered according to the cell-type composition of their 20 nearest neighbors in X-Y space. Two clusters were highly enriched in endocrine cells and were merged to capture islets. One cluster was enriched in B cells, CD8+ T cells, and CD4+ T cells and corresponded to the T/B cell aggregates that we had observed in the raw tissue images.

**Single-Cell (sc) RNA Sequencing and T Cell Receptor (TCR) Sequencing**

Islet isolation

Resected pancreas was collected during a partial pancreatectomy as part of a Whipple procedure. Pancreas tissue was immediately placed into Cold-Storage Solution (Mediatech, Corning) and ice to preserve tissue integrity. Pancreas tissue was cleaned of fat and connective tissue and then decontaminated for five minutes in a cold, 10° C, Cefazolin/Gentamycin solution prior to enzyme distention with cold GMP Collagenase NB1 (Nordmark), 5.3 Wunsch Units/mL, and GMP Neutral Protease NB (Nordmark), 0.64 DMC Units/mL, solution in a volume relative to the mass of pancreas. Cold enzyme solution was injected into each piece of pancreas using a 10 mL syringe and a 21G needle until pancreas was fully distended. Pancreas was placed into a Ultem 125 mL Ricordi Chamber (BioRep), containing silicon nitride marbles and a 533 µm wire mesh. Digestion circuit was filled with Phase I solution (Mediatech, Corning), heated to 37° C, and allowed to digest for 16 minutes. Presence of islets, within the pancreatic digest, was confirmed using a zinc-chelating dye, Dithizone (Sigma). Digestion was stopped by cooling and diluting digested tissue with supplemented cold RPMI 1640 media (Mediatech, Corning). Diluted digest was then centrifuged at 170 *g* for 4 minutes and tissue pellets were washed again in a Cold-Storage solution and centrifuged, prior to pellets being placed into CRML 1066 culture media (Mediatech, Corning) containing human serum albumin, DNAse, and cirpofloxacin. Free islets were hand-picked using inverted microscope and 200 µL pipet. Islets were then further dissociated for 30 minutes at 37° C in an enzyme-free, PBS-based, Cell Dissociation buffer (Life Technologies). Islets were thoroughly resuspended and filtered using a 40 µm strainer over a flow cytometry tube, before centrifugation at 1400 rpm for 4 minutes and resuspension for 10X scRNA and TCR sequencing.

Pancreatic Tumor digestion

For the pancreatic adenocarcinoma, a section of freshly isolated pancreatic tumor was provided following surgical excision by a pathologist. Pancreatic tumor was minced and digested for one hour with a buffer consisting of RPMI media supplemented with 20 mg/mL DNase I (Roche), and 125 U/mL collagenase D (Roche) and homogenized using a 40 µm cell strainer to prepare single cell suspensions.

Peripheral Blood Mononuclear Cell (PBMC) isolation

PBMCs were collected at the same time as the Whipple surgery was performed with isolation from a Ficoll density gradient. Some cells were immediately utilized in a single-cell suspension for 10X single-cell RNA and TCR sequencing while others were cryopreserved in 10% dimethyl sulfoxide (DMSO) and 90% fetal bovine serum (FBS).

10X scRNA and TCR sequencing

For 10X Genomics scRNA and TCR sequencing, all cells recovered from the islets, 20,000 cells from the PBMC preparation, and viable (determined by LIVE/DEAD blue staining) flow cytometry sorted cells from the pancreatic tumor were isolated and then processed individually by the Chromium Single Cell V(D)J Enriched and 5′ Gene Expression library generation and sequencing following manufacturer’s instructions. Cells were loaded onto a Chromium Next GEM chip G. Cells were lysed for reverse transcription and cDNA amplification in the Chromium Controller (10X Genomics). Full-length cDNA along with cell barcode identifiers were PCR amplified, and sequencing libraries were prepared and normalized. The constructed library was sequenced on NovaSeq S4 flow cell (Illumina). Library preparation and sequencing was performed at the Institute for Human Genetics at the Parnassus campus of the University of California San Francisco.

scRNA-seq and TCR-seq data processing

Cell Ranger Single-Cell Software Suite (version 3.1.0, 10X Genomics) was used for library demultiplexing, FASTQ file generation, read alignment, doublet filtering, barcode counting, unique molecular identifier (UMI) counting, and to generate feature barcode matrices, determine clusters, and perform gene expression analysis. Droplet-based sequencing data were aligned and quantified against the GRCh38 human reference genome. Quality of cells from each sample was then assessed based on the number of total UMI counts per cell (library size), the number of detected genes per cell, and the proportion of mitochondrial gene counts with cells then retained for downstream analysis.

scRNA-seq data analysis

scRNA sequencing analysis for islets, PBMCs, and pancreatic tumor cells was performed using the Seurat package(10–13). First, quality control measures were implemented: features were selected if more than 3 cells shared that gene (“feature”), and cells had to have at least 200 features. Cells were then selected that had less than 10% mitochondrial gene signatures and less than 60% ribosomal gene signatures. SCTransform was used on each respective cell compartment (islet, PBMC, pancreatic tumor) and then they were integrated on 3000 features using SelectIntegrationFeatures, PrepSCTIntegration, FindIntegrationAnchors and IntegrateData. Next, we used the Azimuth reference mapping approach to identify clusters (through RunAzimuth aligning to the human PBMC reference database)(10,11). The islet cell cluster was manually identified by the presence of glucagon (*GCG* gene*)* and synaptophysin (*SYP* gene). Separately, filtered cell clusters from the islet sample were manually annotated to better characterize non-immune cells within this sample that are poorly represented in the Azimuth PBMC reference data. Cell annotations used canonical markers of pancreatic cell types and the Azimuth pancreas reference data for pancreas annotation and canonical markers of T cell and B cell subsets (i.e. memory and naïve subsets) for immune markers(10,14,15). FindAllMarkers was used to identify differentially expressed genes within each cluster. Top genes were then compared to canonical cell markers to identify clusters (Figure S2).

scTCR-seq data analysis

scTCR-seq V(D)J analysis for islets, PBMCs, and pancreatic tumor cells was performed using the Seurat package. For cells with multiple TCR beta chain reads, the highest read was selected. Cells without TCR beta chain data were excluded from the analysis. Shared and expanded TCRs were annotated based on enrichment of the CDR3β sequence. The nPOD JDRF database maintained by Maki Nakayama were matched to islet, PBMC and pancreatic tumor TCR beta chains, by restricting to the CDR3β sequence. TCR beta chains from the McPAS-TCR database (http://friedmanlab.weizmann.ac.il/McPAS-TCR/)(16) annotated to have been isolated from individuals with T1D were compared to CDR3β sequence from the patient with CPI-D. Exact matches and matches with a maximum Levenshtein distance (LD) of one between CDR3β sequence are shown. The Levenshtein distance is the edit distance between two sequences, such as the sum of insertions, deletions or substitutions which allows for identification of imperfectly shared CDR3β sequences. Features of interest were quantified, including the number and frequency of shared CDR3β sequences between compartments (islets, pancreatic tumor, and PBMCs), expanded (as defined by ≥2 TCRs) and matched CDR3β sequences between the CPI-D patient and T1D TCR databases, which were compared across different T cell subsets identified through cluster annotation as detailed above. The McPAS-TCR database was also queried for proposed antigen recognition for T1D, which was manually collated from published literature. Matched CDR3β sequences are annotated in Supplemental Table 1.

**Supplementary Figure Legends**

**Supplementary Figure 1. Details of methodologies used to interrogate tissue and blood from a CPI-D patient.**

**(A)** Methodologies used to assess the immune microenvironment in the pancreas, including both whole tissue and islet-specific, PBMCs and pancreatic tumor of the CPI-D patient. **(B)** Markers used in the CODEX panel to classify cell types in the pancreatic tissue. **(C)** Regions of the pancreatic tissue imaged by CODEX.

**Supplementary Figure 2. Characterization of islet-derived immune infiltrate from a CPI-D patient.**

**(A)** UMAP of cells recovered from scRNA-seq of islets and with cluster identification through canonical gene annotation by the Azimuth reference dataset for islets. **(B)** Counts of cells within each cluster. **(C)** Feature plots of canonical genes used for cluster identification are projected onto the UMAP with gene expression in blue (logCPM).

**Supplementary Figure 3. Lymphoid aggregates in the pancreas of individuals with spontaneous T1D.**

The pancreas of two individuals with spontaneous T1D were obtained through the nPOD program. **(A)** Histology of immune infiltrate surrounding an islet is shown by H&E (far left panel) and staining with glucagon (denoted as GCG for alpha cells), insulin (denoted as INS for beta cells), CD3 or CD8 (T cells), and CD20 (B cells). These lymphoid aggregates were present adjacent to islets both with and without insulin (INS) alongside glucagon (GCG). nPOD patient ID, diabetic status, and time from diagnosis for which the pancreas was received by nPOD is shown. Scale bar 200 µm.

**Supplementary Figure 4. Expression of immunoregulatory markers in T/B cell clusters from the pancreas of the CPI-D patient and individuals with spontaneous T1D.** Mean fluorescence intensity (MFI) of immunomodulatory markers determined by CODEX expressed in CD4+FOXP3+ T cells, CD4+FOXP3- T cells, CD8+ T cells and B cells in the pancreas from a CPI-D patient in **(A)** T/B cell clusters and **(B)** non-T/B cell cluster regions. A paralleled assessment was also performed for eight pancreata from individuals who were diagnosed with spontaneous T1D acquired from nPOD(3) in **(C)** T/B cell clusters and **(D)** non-T/B cell cluster regions.

**Supplementary Figure 5. Enriched gene expression of germinal center features in B cells from the islet compared to periphery of a CPI-D patient. (A)** Cell density of CD138+ cells in T/B cell clusters from individuals with spontaneous T1D compared to CPI-D, the latter of which they were not detected (n.d.) as measured with CODEX, with spontaneous T1D cases re-analyzed from Barlow *et al.* (3). T/B cell clusters were absent in case 6323 and was therefore not included in the analysis of CD138+ cells. **(B)** Annotation of B cell subsets from islets and PBMCs from CPI-D patient were defined by aligning scRNA-seq to the Azimuth reference(10). **(C)** Identification of feature marker expression in B cells aligned with cellular subtypes and function(14,15).

**Supplemental Figure 6. Characterization of T cell subsets associated with shared, matched or expanded CDR3β sequences from a CPI-D patient.**

**(A)** Annotation of T cell subsets isolated from islets, PBMCs and pancreatic tumor of a CPI-D patient. T cell subsets were identified by aligning scRNA-seq to the Azimuth reference(10,11). **(B)** CDR3β sequences that are either shared between compartments (islets, PBMCs, tumor) or expanded within each compartment (as defined by ≥2 CDR3β sequences), both or neither are shown. **(C)** CDR3β sequences that were identified to match existing T1D TCR databases from McPAS-TCR or the nPOD program. McPAS-TCR data was matched on either exact matches or within 1 Levenshtein distance (LD), as specified.

**References**

1. Campbell‐Thompson M, Wasserfall C, Kaddis J, Albanese‐O’Neill A, Staeva T, Nierras C, et al. Network for Pancreatic Organ Donors with Diabetes (nPOD): developing a tissue biobank for type 1 diabetes. Diabetes Metab Res Rev. 2012 Oct 2;28(7):608–17.

2. Pugliese A, Yang M, Kusmarteva I, Heiple T, Vendrame F, Wasserfall C, et al. The Juvenile Diabetes Research Foundation Network for Pancreatic Organ Donors with Diabetes (nPOD) Program: goals, operational model and emerging findings. Pediatr Diabetes. 2014 Feb 10;15(1):1–9.

3. Barlow GL, Schürch CM, Bhate SS, Phillips DJ, Young A, Dong S, et al. The extra-islet pancreas supports autoimmunity in human type 1 diabetes. Elife [Internet]. 2025 Apr 15;13. Available from: https://elifesciences.org/articles/100535

4. Black S, Phillips D, Hickey JW, Kennedy-Darling J, Venkataraaman VG, Samusik N, et al. CODEX multiplexed tissue imaging with DNA-conjugated antibodies. Nat Protoc. 2021 Aug 2;16(8):3802–35.

5. Schürch CM, Bhate SS, Barlow GL, Phillips DJ, Noti L, Zlobec I, et al. Coordinated Cellular Neighborhoods Orchestrate Antitumoral Immunity at the Colorectal Cancer Invasive Front. Cell. 2020 Sep;182(5):1341-1359.e19.

6. Kennedy‐Darling J, Bhate SS, Hickey JW, Black S, Barlow GL, Vazquez G, et al. Highly multiplexed tissue imaging using repeated oligonucleotide exchange reaction. Eur J Immunol. 2021 May 10;51(5):1262–77.

7. Goltsev Y, Samusik N, Kennedy-Darling J, Bhate S, Hale M, Vazquez G, et al. Deep Profiling of Mouse Splenic Architecture with CODEX Multiplexed Imaging. Cell. 2018 Aug;174(4):968-981.e15.

8. Lee MY, Bedia JS, Bhate SS, Barlow GL, Phillips D, Fantl WJ, et al. CellSeg: a robust, pre-trained nucleus segmentation and pixel quantification software for highly multiplexed fluorescence images. BMC Bioinformatics. 2022 Dec 18;23(1):46.

9. Bhate SS, Barlow GL, Schürch CM, Nolan GP. Tissue schematics map the specialization of immune tissue motifs and their appropriation by tumors. Cell Syst. 2022 Feb;13(2):109-130.e6.

10. Hao Y, Hao S, Andersen-Nissen E, Mauck WM, Zheng S, Butler A, et al. Integrated analysis of multimodal single-cell data. Cell. 2021 Jun;184(13):3573-3587.e29.

11. Stuart T, Butler A, Hoffman P, Hafemeister C, Papalexi E, Mauck WM, et al. Comprehensive Integration of Single-Cell Data. Cell. 2019 Jun;177(7):1888-1902.e21.

12. Butler A, Hoffman P, Smibert P, Papalexi E, Satija R. Integrating single-cell transcriptomic data across different conditions, technologies, and species. Nat Biotechnol. 2018 May 2;36(5):411–20.

13. Satija R, Farrell JA, Gennert D, Schier AF, Regev A. Spatial reconstruction of single-cell gene expression data. Nat Biotechnol. 2015 May 13;33(5):495–502.

14. Fitzsimons E, Qian D, Enica A, Thakkar K, Augustine M, Gamble S, et al. A pan-cancer single-cell RNA-seq atlas of intratumoral B cells. Cancer Cell. 2024 Oct;42(10):1784-1797.e4.

15. Yang SY, Long J, Huang MX, Luo PY, Bian ZH, Xu YF, et al. Characterization of Organ-Specific Regulatory B Cells Using Single-Cell RNA Sequencing. Front Immunol. 2021 Sep 14;12.

16. Tickotsky N, Sagiv T, Prilusky J, Shifrut E, Friedman N. McPAS-TCR: a manually curated catalogue of pathology-associated T cell receptor sequences. Bioinformatics. 2017 Sep 15;33(18):2924–9.
