# Supplementary material for: Autoimmune origin for immune checkpoint inhibitor-diabetes revealed by deep immune phenotyping of the pancreas
Source: J Immunother Cancer. 2025 Aug 14;13(8):e011818. doi: 10.1136/jitc-2025-011818 (PMC12359507; doi:10.1136/jitc-2025-011818)

Supplementary Figure 1

A

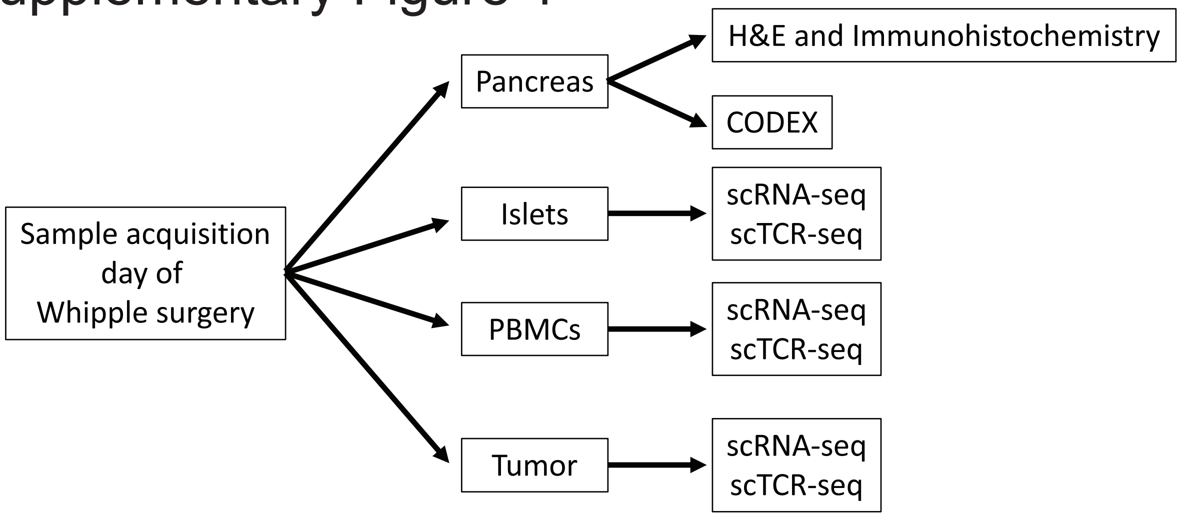

B

LYMPHOID	MACROPHAGE	FUNCTIONAL	TISSUE	
BCL-2	Galectin-3	Ki67	Chromogranin A	S100A6
FOXP3	CD16	TOX	Proinsulin	Cytokeratin
CD8	CD11c	Lag3	PDX1	Alpha-SMA
CD45RA	CD163	PD-1	Glucagon	CD45
CD3	CD206	CD69	Insulin	HABP
CD20	HLA-DR	ICOS	MUC-1	Draq 5
CD4	VISTA	Granzyme B	Somatostatin	HOECHST
CD57	CD68	OX40	Synaptophysin	
CD56		IDO	CollIV	
CD44			NaKATPase	
CD45RO	**GRANULOCYTE**		HLA-ABC	
TCR g/d	CD15		Podoplanin	
CD138	MPO		CD31	

C

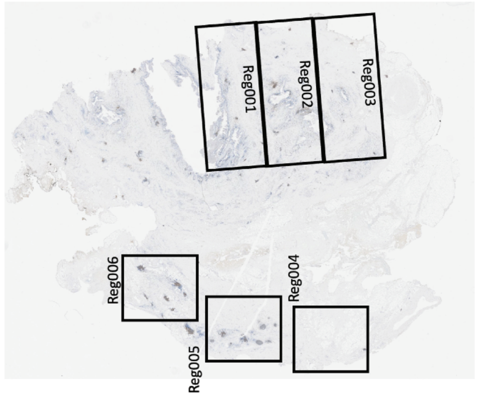

Supplement: online supplemental file 3 [file jitc-13-8-s003.pdf]
