# Supplementary figures and images for: Autoimmune origin for immune checkpoint inhibitor-diabetes revealed by deep immune phenotyping of the pancreas
Source: J Immunother Cancer. 2025 Aug 14;13(8):e011818. doi: 10.1136/jitc-2025-011818 (PMC12359507; doi:10.1136/jitc-2025-011818)

# Supplementary Figure 2

A

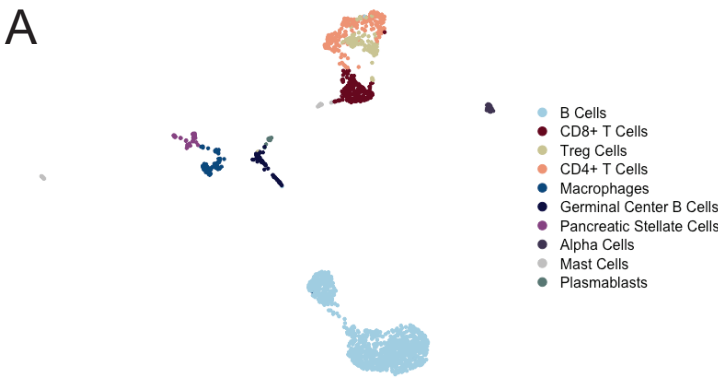

B

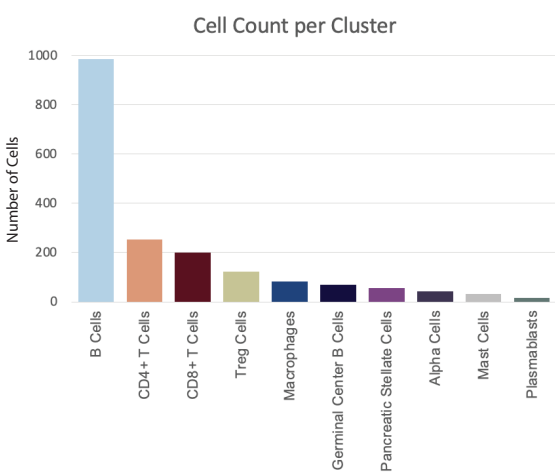

C

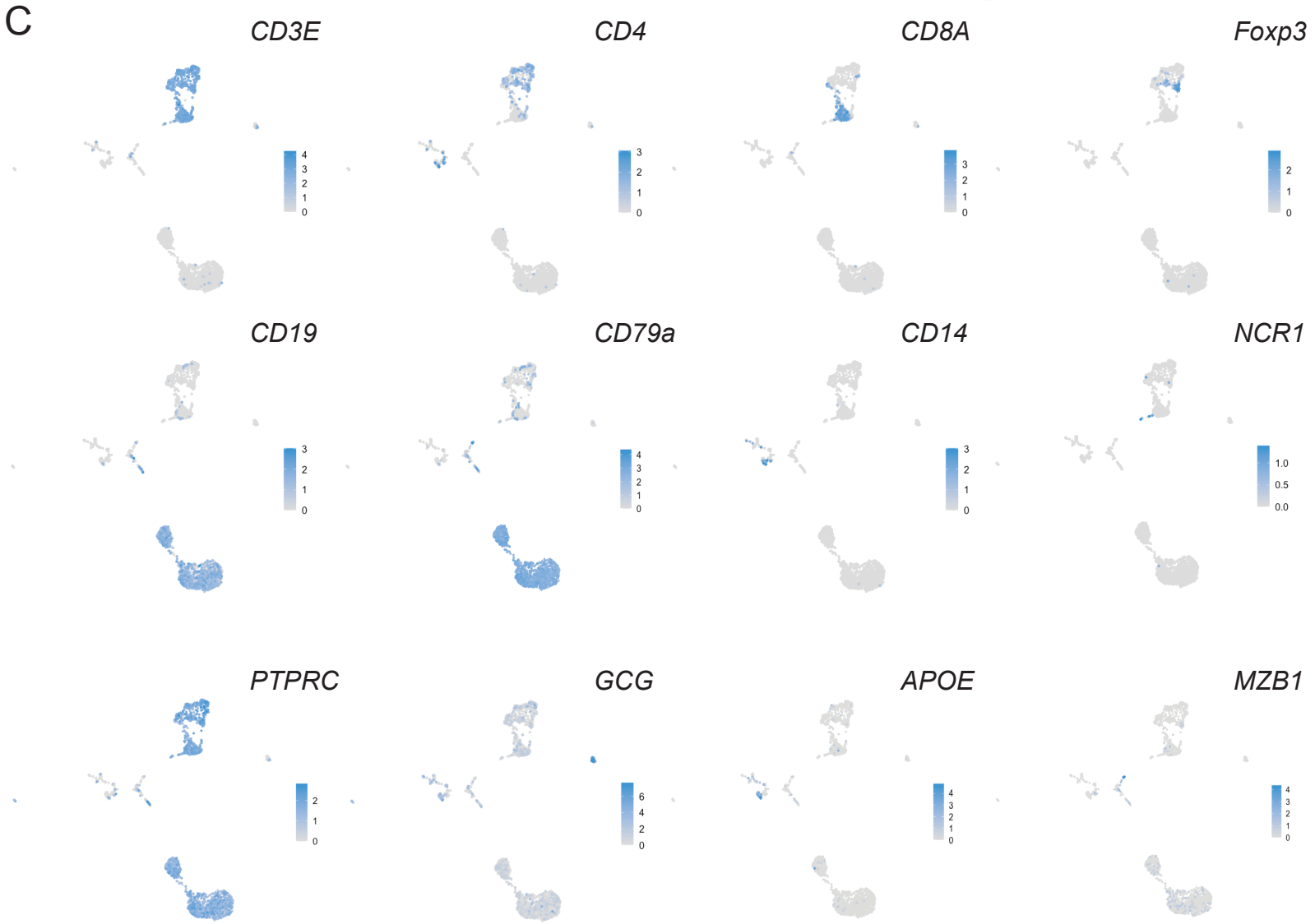

Supplement: online supplemental file 4 [file jitc-13-8-s004.pdf]

# Supplementary Figure 3

A

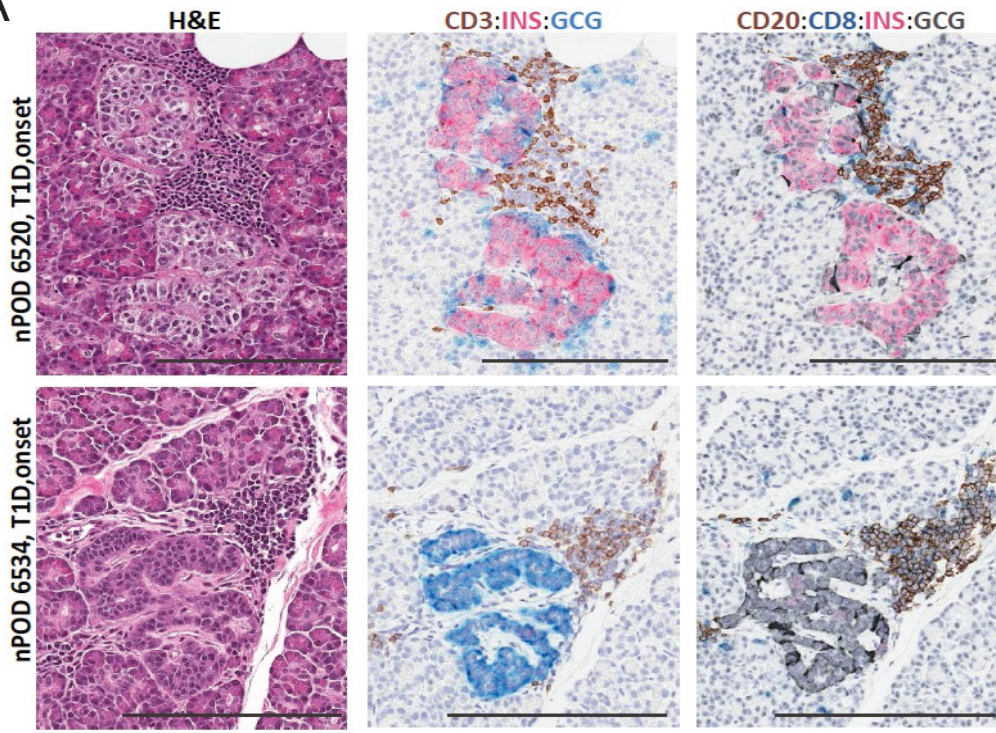

Supplement: online supplemental file 5 [file jitc-13-8-s005.pdf]

# Supplementary Figure 4

A

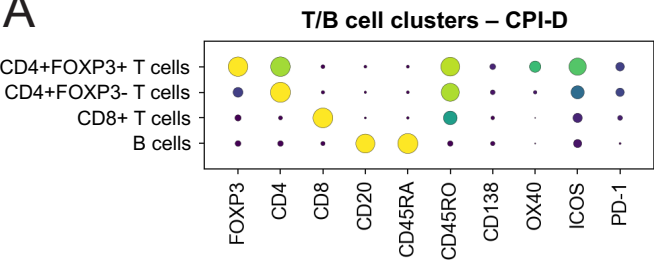

B

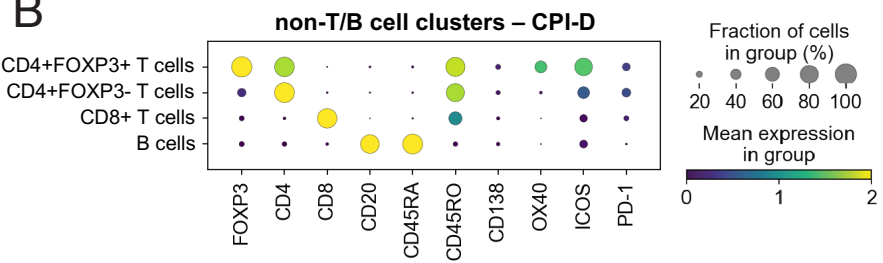

C

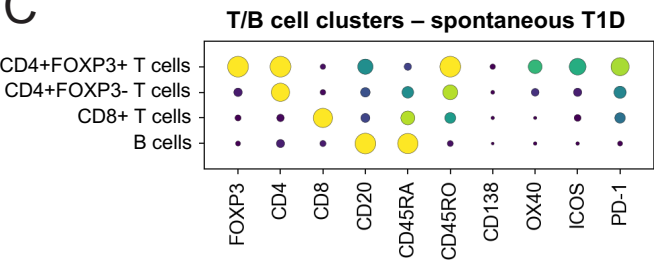

D

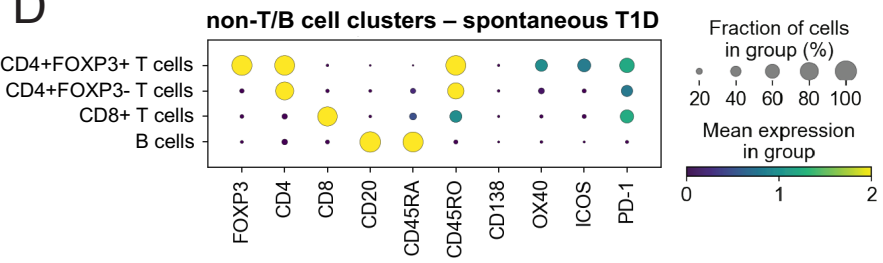

Supplement: online supplemental file 6 [file jitc-13-8-s006.pdf]

# Supplementary Figure 5

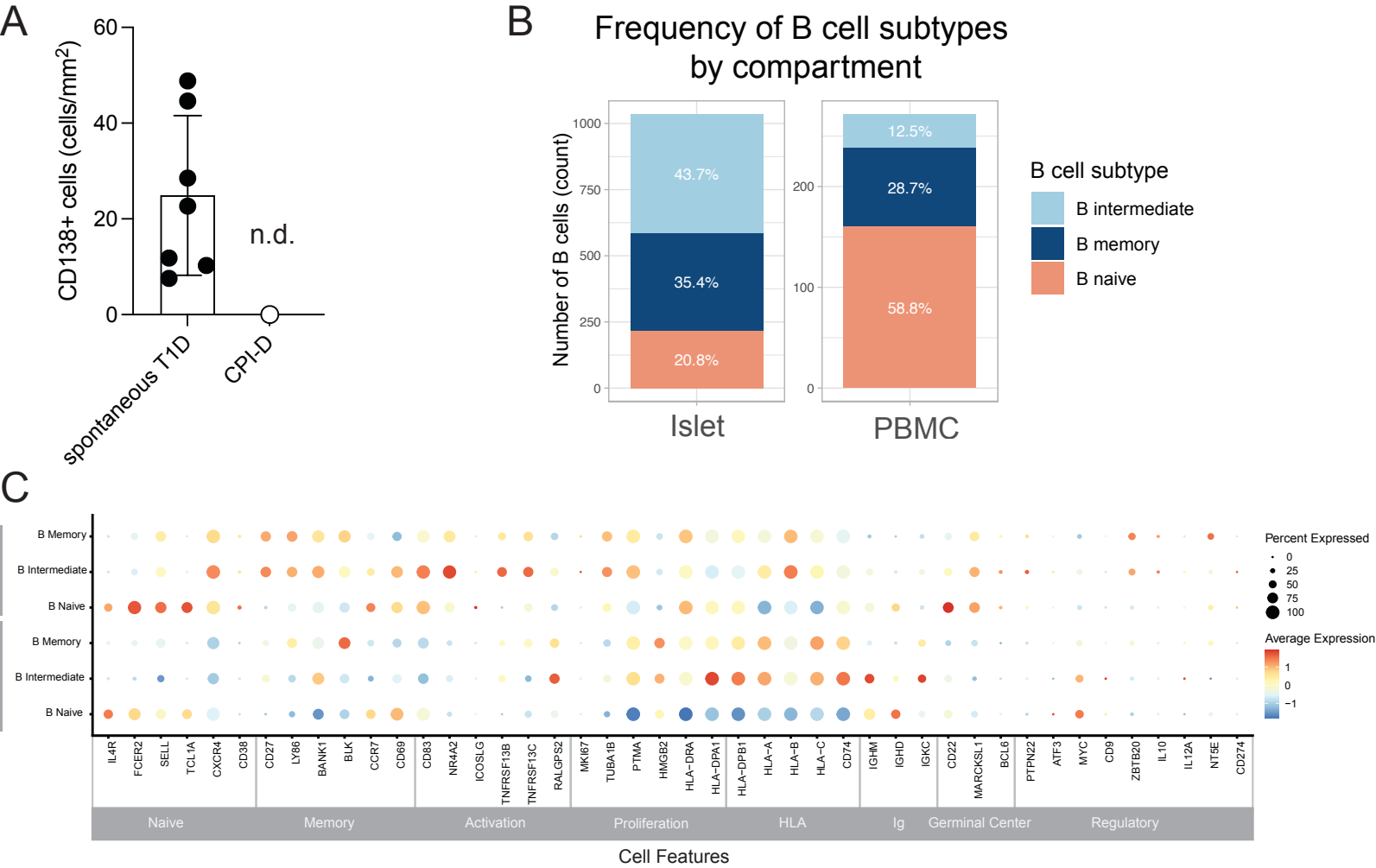

Supplement: online supplemental file 7 [file jitc-13-8-s007.pdf]

# Supplementary Figure 6

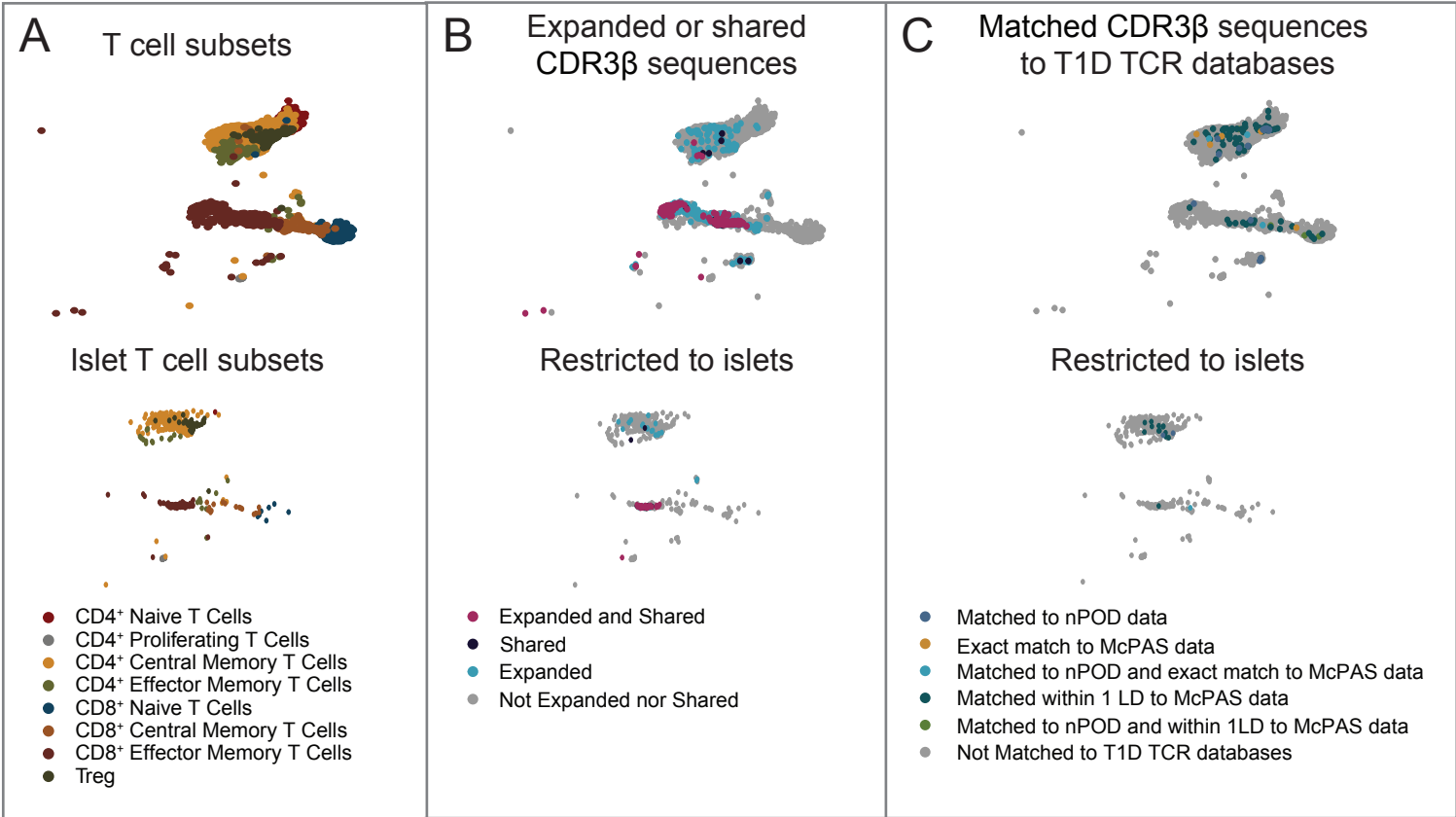

Supplement: online supplemental file 8 [file jitc-13-8-s008.pdf]
